# Host-Associated Biofilms: *Vibrio fischeri* and Other Symbiotic Bacteria Within the Vibrionaceae

**DOI:** 10.3390/microorganisms13061223

**Published:** 2025-05-27

**Authors:** Joaquin Lucero, Michele K. Nishiguchi

**Affiliations:** Department of Molecular and Cell Biology, University of California, Merced, CA 95343, USA; jlucero25@ucmerced.edu

**Keywords:** symbiosis, Vibrionaceae, bioluminescence, Cephalopoda

## Abstract

Biofilm formation is important for microbial survival, adaptation, and persistence within mutualistic and pathogenic systems in the Vibironaceae. Biofilms offer protection against environmental stressors, immune responses, and antimicrobial treatments by increasing host colonization and resilience. This review examines the mechanisms of biofilm formation in *Vibrio* species, focusing on quorum sensing, cyclic-di-GMP signaling, and host-specific adaptations that influence biofilm structure and function. We discuss how biofilms differ between mutualistic and pathogenic species based on environmental and host signals. Recent advances in omics technologies such as transcriptomics and metabolomics have enhanced research in biofilm regulation under different conditions. Horizontal gene transfer and phase variation promote the greater fitness of bacterial biofilms due to the diversity of environmental isolates that utilize biofilms to colonize host species. Despite progress, questions remain regarding the long-term effects of biofilm formation and persistence on host physiology and biofilm community dynamics. Research integrating multidisciplinary approaches will help advance our understanding of biofilms and their implications for influencing microbial adaptation, symbiosis, and disease. These findings have applications in biotechnology and medicine, where the genetic manipulation of biofilm regulation can enhance or disrupt microbiome stability and pathogen resistance, eventually leading to targeted therapeutic strategies.

## 1. Introduction

A biofilm is a structured community of microorganisms that adhere to surfaces and are embedded within a self-produced matrix of extracellular polymeric substances, or EPSs. These microbial communities are highly organized and can be found in a wide range of environments. Biofilms play a pivotal role in the survival and persistence of many microorganisms, including both beneficial and pathogenic species. They are crucial for the establishment of symbiotic relationships between microbes and their hosts. In marine ecosystems, biofilms are integral to the establishment of host–microbe interactions, from nutrient exchange within the host to protection against environmental stressors. This review explores the significance of biofilms in a variety of symbiotic species within the Vibrionaceae. By examining biofilm-forming mechanisms in *Vibrio fischeri* and its relatives, we will discuss the ecological and evolutionary significance of biofilms to broaden our understanding of biofilm formation across distinct species of *Vibrio* and *Photobacterium* in host-associated contexts.

**Figure 1:**
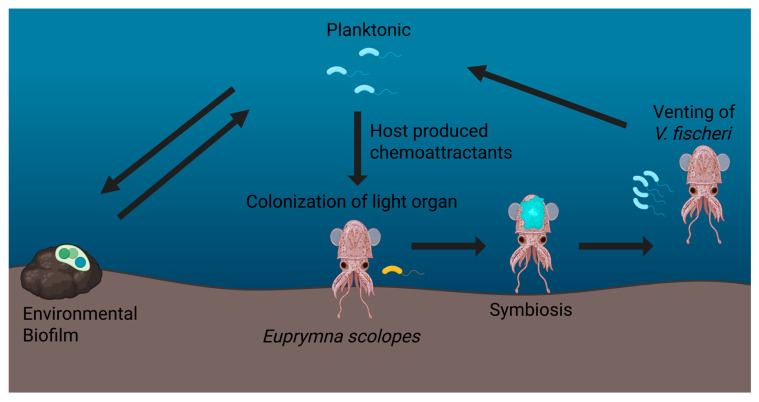
Transition between environmental and squid host-associated *Vibrio fischeri. V. fischeri* exists in both environmental biofilms and a planktonic (free-living) state. Host-produced chemo-attractants recruit planktonic *V. fischeri* to the squid’s light organ, where a series of specific biochemical filters only allow symbiosis-competent *V. fischeri* to colonize. After colonization, *V. fischeri* can then establish a dense community or biofilm, allowing the production of bioluminescence through quorum sensing. Once bioluminescence is no longer needed for nocturnal counterillumination and with the onset of daylight, the host squid vents 95% of its symbiont population at dawn, releasing competent *V. fischeri* back into the environment. These free-living *V. fischeri* may form biofilms on other abiotic substrates or colonize new juvenile squids, continuing the cycle between free-living and host-associated life histories.

One example of how biofilms provide a beneficial asset to the host are those found in sepiolid squids (Cephalopoda: Sepiolidae) and their bioluminescent symbionts in the genera *Vibrio* and *Photobacterium.* This association represents a tractable model system for studying the physiological, biochemical, and molecular mechanisms of animal host–microbe interactions. *V. fischeri* utilizes biofilm formation as a mechanism for host colonization, providing the squid with bioluminescence via quorum sensing, while benefiting from the nutrient-rich environment of the host light organ Figure 1 [1]. Upon hatching, juvenile squids acquire *V. fischeri* from the surrounding seawater through a selective and targeted process. The bacteria then migrate toward and colonize the crypts of the squid’s light organ [1]. In the nutrient-rich light organ, squids use *V. fischeri* for counterillumination during the night, a behavior that allows them to match downwelling moonlight and starlight, rendering them less visible to predators and prey below [2]. Biofilm formation enables *V. fischeri* to adhere to host epithelial surfaces in the light organ, grow rapidly, and establish a stable, self-sustaining population that replenishes itself each morning through a venting process with the onset of dawn. During this diurnal cycling, *V. fischeri* biofilm production is tightly regulated by a variety of genetic and environmental factors, most notably the quorum-sensing system LuxIR, which coordinates bacterial behavior in response to population cell density when cells reach the late logarithmic phase right before dusk [3,4]. Additionally, the bacterium modulates its behavior in response to host-derived signals, such as nitric oxide and other immune factors, allowing it to adapt to the host light organ’s ever-changing internal environment [3,5,6]. These biofilms not only enable attachment and colonization but also play crucial roles in protecting *V. fischeri* from host immune responses and environmental challenges, fostering a stable and persistent symbiosis [3,4,5,7,8,9].

However, the biofilm-forming capabilities of *V. fischeri* are not unique in the microbial world. Other *Vibrio* species, such as *V. cholerae* and *V. vulnificus*, as well as *Photobacterium* species, exhibit diverse biofilm-forming strategies that enable them to thrive in both pathogenic and mutualistic states [10,11]. In the case of *V. cholerae*, biofilm formation is essential for its ecological persistence and successful colonization of the human intestine, with biofilm structures enhancing resistance to environmental stressors and immune responses [12,13]. Similarly, *V. vulnificus*, a known marine pathogen, forms biofilms on both biotic and abiotic surfaces, playing a critical role in its pathogenicity and persistence in marine and clinical environments. In contrast, *Photobacterium* species, which form beneficial relationships with marine hosts, produce biofilms that enhance stability and facilitate bioluminescence. This results in improved host camouflage and symbiont survival under extreme deep-sea conditions such as high hydrostatic pressure and low nutrient availability, ultimately contributing to the ecological fitness of both partners [13,14]. Other marine bacteria such as *Roseobacters* also form biofilms and have essential roles in colonizing the accessory nidamental gland by coating the egg surface of *Euprymna scolopes* to protect against fouling and infection [15,16]. They do not exhibit the bioluminescent mutualism or crypt-specific colonization strategies seen in *V. fischeri***.** Such studies of biofilm formation in *Vibrio* and *Photobacterium* species have far-reaching implications for understanding microbial ecology, evolution, and persistence throughout various habitats [17,18,19].

Biofilms are a critical component of microbial life in diverse habitats, especially in marine ecosystems, where they influence ecosystem health, resilience, and function. Marine biofilms play an essential role in nutrient cycling, contributing to the regeneration of organic matter, nitrogen fixation, and other biogeochemical processes. For instance, biofilms in coral reefs, seagrasses, and oyster beds mediate the cycling of nitrogen and other nutrients, thus supporting primary productivity and maintaining the stability of these vital ecosystems [20]. In these habitats, biofilms also help protect microbial communities from external stressors such as ocean acidification and warming by creating a physical barrier that buffers temperature fluctuations, concentrating stress-response molecules, and facilitating cooperative interactions that enhance thermal tolerance [21]. Biofilms are also integral to microbial survival, contributing to colonization, immune evasion, and nutrient exchange in many animal mutualisms, where all organisms involved benefit. For example, biofilms formed by coral-associated *Vibrio* species play a complex role, simultaneously supporting beneficial associations when the host is healthy while contributing to pathogenesis and coral disease under stressful conditions such as prolonged exposure to high temperatures [22]. Understanding the delicate balance between mutualistic and pathogenic life histories of biofilms is critical for addressing global challenges such as coral bleaching, which are impacted by climate change [23].

Biofilm research offers valuable insights into microbial adaptation, evolutionary dynamics, and pathogenesis. In *Vibrio* species, biofilm formation plays a central role in ecological persistence, host colonization, and adaptation to fluctuating environments. The protective and structured nature of biofilms enables *Vibrio* to tolerate osmotic stress and resist antimicrobial agents, mirroring mechanisms seen in other pathogenic bacteria [21,22,23]. Close bacterial proximity within biofilms facilitates horizontal gene transfer, including genes for virulence and resistance, which has been observed in diverse *Vibrio* populations and contributes to their ecological versatility [22,23,24,25]. In *V. fischeri*, biofilm formation is essential for successful symbiosis with squid hosts, and experimental evolution studies have shown that biofilms support adaptation to novel environmental pressures such as salinity and temperature shifts, as well as novel host species [26,27].

While most research on *Vibrio* biofilms centers on marine ecology and symbiosis, emerging studies have begun to explore their potential applications. Some *Vibrio* species, such as *V. alginolyticus* and *V. natriegens*, have shown promise in biofilm-based bioremediation efforts due to their ability to degrade pollutants in marine environments [28,29], and marine biofilms, including those formed by *Vibrio*, are increasingly recognized as valuable sources of antimicrobial peptides with biotechnological potential [24]. Although *Vibrio* biofilms have not been widely implicated in chronic wound infections, unlike *Pseudomonas* or *Staphylococcus*, certain pathogenic strains like *V. vulnificus* and *V. parahaemolyticus* are known to form biofilms on medical devices and surfaces, contributing to infection persistence [30,31]. Understanding *Vibrio* biofilm formation in both beneficial and pathogenic contexts enhances our ability to engineer microbial systems for biotechnology and to design effective strategies against biofilm-associated infections [30,31,32].

## 2. Biofilms: Essential Structures in Symbiosis

Biofilms play a pivotal role in facilitating host–microbe interactions during symbiosis. By forming biofilms, mutualistic bacteria like *V. fischeri* can adhere to host tissues, resist abiotic stresses, and establish long-term associations with their host [25]. Biofilms are crucial in the colonization of the Hawaiian bobtail squid *Euprymna scolopes*, as they enable *V. fischeri* to populate the squid’s light organ and maintain bioluminescence as well as reduce immune responses from the host [1,26]. *Photobacterium* species form biofilms on fish skin (broad host range) and within fish light organs (species-specific association), and only those associated with light organ-containing hosts contribute to host protection against predators or communication through bioluminescence [27]. Within the light organ, beneficial bacteria recycle host-derived nutrients such as amino acids and peptides while producing bioluminescence that benefits the host [28]. These interactions are also influenced by a variety of environmental factors and host-derived signaling that regulates biofilm development.

Environmental conditions, including light exposure [29,30], and host-derived signals, such as nitric oxide and antimicrobial peptides, regulate biofilm formation in *V. fischeri*, influencing bacterial stress responses, gene expression, and biofilm architecture [18,31]. These dynamic interactions create a feedback loop between *V. fischeri* and its host by fine-tuning biofilm growth to optimize *V. fischeri* under such varying environmental conditions. For example, the light-regulated LuxIR signal integrates with the AinS quorum-sensing pathway to modulate biofilm structure and density, allowing the bacteria to adjust their attachment and dispersal in response to population density and host signals [32]. Host-secreted nutrients regulate bacterial metabolism, exopolysaccharides, and the adhesion necessary for biofilm stability [33,34]. Signaling via H-NOX regulators controls bacterial gene expression, influencing stress responses and contributing to the regulation of colonization in symbiotic *V. fischeri* [35]. Together, these regulatory systems impose an adaptive response that allows *V. fischeri* to establish and maintain a stable and dynamic mutualistic association with bobtail squids.

## 3. *Vibrio fischeri* as a Model for the Study of Host-Associated Biofilms

Additionally, symbiosis between *V. fischeri* and the Hawaiian bobtail squid *E. scolopes* (Cephalopoda: Sepioliadae) serves as a hallmark model for studying the molecular mechanisms of biofilm regulation and production [3]. Quorum sensing is central to biofilm formation and symbiosis in *V. fischeri*. The LuxIR system mediates the cell-density-dependent regulation of bioluminescence and biofilm maturation [36]. As bacterial populations grow, the autoinducer N-acyl-homoserine lactone accumulates, triggering the activation of genes necessary for biofilm and light production and other group behaviors such as motility and virulence [37,38]. Additionally, the *syp* symbiosis polysaccharide locus regulates the synthesis of extracellular matrix components essential for biofilm stability and structural integrity [39]. Mutations in genes such as *rscS* and *sypG* disrupt *syp* locus activation, impairing biofilm production and reducing the bacterium’s ability to establish stable biofilms with the host [39,40].

Beyond quorum sensing, colonization is also influenced by additional molecular regulators such as genes controlling chemotaxis and adherence [41]. For example, Clp has elevated expression which is often linked to better biofilm formers and more successful colonizers in *V. fischeri* biofilms [18]. *sypF*, a hybrid sensor kinase, coordinates biofilm formation via upstream response regulators that control pili expression and function that are essential for effective colonization [42]. Symbiotic loci such as *pilA*, *flaA*, and *mshA* are important for *V. fischeri* to locate, colonize, and attach to the host light organ. For example, *flaA* mutations result in impaired motility and a reduction in colonization efficiency, which highlights the importance of motility-related genes for successful symbiosis [43] *V. fischeri* uses flagellar motility and pili to navigate chemical gradients in the host, such as those produced by chitin oligosaccharides, and to help them attach firmly to host tissues [44,45]. Surface adhesins, including those encoded by type IV *pil* and *msh* genes, further mediate bacterial attachment and biofilm stabilization [9]. For example, the *pil* operon in *V. fischeri* (*pilABCD*) encodes for components of type IV pili, which produce a protein essential for attachment and persistence. Molecular differences in *pilB* and *pilD* loci found in different *V. fischeri* strains suggest that these genes are critical for host recognition and specificity. Swapping pili genes from different *V. fischeri* strains demonstrates how such subtle differences in these loci are responsible for native versus non-native strain specificity [7,18,25].

To facilitate the synthesis of the genetic and regulatory components discussed above, Table 1 summarizes key genes and host-derived factors involved in biofilm formation and colonization in *V. fischeri*.

## 4. Expanding the Lens: Other *Vibrio* and *Photobacterium* Species

While *V. fischeri* serves as an exemplary model for biofilms during symbiosis, other *Vibrio* species and related bacteria exhibit distinct biofilm strategies that highlight the versatility of biofilm-mediated interactions. Comparative studies on *V. cholerae*, *V. vulnificus*, and *Photobacterium* species reveal a wide array of biofilm mechanisms that contribute to their ecological success [58]. For instance, *V. cholerae* biofilms are crucial for environmental persistence and host pathogenicity, utilizing exopolysaccharides and extracellular DNA to enhance surface attachment and microbial resilience [59,60]. These biofilms facilitate *V. cholerae* survival in aquatic environments and transition to human hosts, playing an important role in its transmission and virulence [61,62,63]. *V. vulnificus* forms robust biofilms on biotic surfaces such as shellfish and arthropod exoskeletons, establishing stable habitats that support environmental persistence and occasional host association [59,60]. *Photobacterium* species, particularly those associated with deep-sea fish or shrimp, form structured biofilms that support bioluminescence, indicating a functional convergence in biofilm-mediated interactions across *Vibrio* bacteria [27,64,65]. The biofilm matrix in these symbiotic interactions enhances the stability of luminescent bacteria, optimizing light production for host communication (high cell density), predator avoidance, and prey attraction [66,67].

Symbiotic versatility is evident in biofilms across different host environments. While *V. fischeri* establishes biofilms within the squid light organ, other *Vibrio* species colonize diverse hosts, including fish and many types of invertebrates. The variability in host-derived signals, including immune factors, microbial competitors, and nutrient availability, influences bacterial biofilm architecture and persistence differentially [3,68,69]. For example, biofilms in fish-associated *Vibrio* strains often exhibit high resistance to host immune responses, whereas coral-associated *Vibrio* biofilms play a dual role in both mutualistic and pathogenic associations under shifting environmental conditions [70,71,72]. *Vibrio* biofilms aid in nutrient cycling and organic matter decomposition in marine ecosystems. This enhances primary productivity and facilitates microbial interactions that regulate carbon and nitrogen fluxes [22,36,73]. Additionally, interspecies biofilm interactions, such as those observed in coral-associated *Vibrio* communities, can influence ecosystem health and resilience against environmental stressors such as ocean acidification and warming [12,72,73]. For example, *Vibrio* biofilms participate in nitrogen cycling by interacting with other microbial communities that regulate denitrification processes, impacting water quality and ecosystem stability in oyster beds [74].

## 5. Adaptive and Functional Roles of Biofilms in Symbiosis

Biofilms are tightly regulated by genetic and molecular pathways that orchestrate surface attachment, matrix production, and dispersal [75]. Quorum sensing, a cell-density-dependent communication system, plays a central role in regulating bioluminescence and biofilm architecture across several *Vibrio* species [32,59,76,77]. *V. cholerae* utilizes the HapR regulator to modulate biofilm dispersal, balancing attachment with planktonic growth [33]. Additionally, the c-di-GMP signaling pathway integrates environmental cues such as nutrient availability and stress signals that regulate key processes in biofilm formation including matrix production as well as cell adhesion [26,78,79]. Variations in c-di-GMP levels are differentiated amongst *Vibrio* and *Photobacterium* species, contributing to species-specific biofilm strategies and resilience to environmental stressors [26,78]

Biofilms also provide a protective niche that enhances bacterial persistence in different environments by protecting bacteria from environmental stressors, antibiotics, and immune effectors [80,81]. In host-associated biofilms, immune evasion occurs through mechanisms such as the suppression of the innate immune response (inflammation, phagocytosis, and other immune cells) and the modulation of host signaling pathways [82,83,84]. For example, *V. fischeri* biofilms in the squid light organ protect the bacteria from oxidative stress while they simultaneously promote symbiotic colonization [3,69]. However, *V vulnificus* biofilms are adept at resisting phagocytosis and antimicrobial peptides contributing to their virulence [85]. The *V. vulnificus* biofilm matrix contains polysaccharides and other extracellular components that act as a physical barrier to the environment [86]. The dense structure of biofilms protects against antimicrobial peptides, allowing their persistence despite the host immune response [87].

Host-specific adaptations select specific biofilm traits through targeted specialization. Different hosts exert various selective pressures on their symbiotic bacteria that influence the genetic makeup of associated *Vibrio* species. With the development of whole-genome sequencing and genome-wide association studies, it has been revealed that *Vibrio* species associated with different hosts exhibit variations in biofilm-associated gene clusters, which are involved in the production of exopolysaccharide and structural components essential for biofilm formation [87,88]. For example, pathogenic *Vibrio* species may acquire additional genes or operons that enhance exopolysaccharide production, allowing the formation of biofilms that are more robust and resistant to the host immune response [89]. Mutualistic strains may lose certain genes involved in biofilm production and regulation, relying instead on certain host-derived factors such as nutrients or signaling molecules to stabilize biofilms [90]. These genetic adaptations are driven by host-specific immune factors and competition amongst other microbes in the surrounding environment [91,92].

In *E. scolopes*–*Vibrio* mutualism, the molecular mechanisms of biofilm regulation have been well studied, but there are still areas that are not well understood. Since biofilm formation is essential for colonization and persistence, any subtle changes can be either beneficial or detrimental to the fitness of symbionts [3,92]. Advancements in omics-based technologies facilitate the study of biofilm regulation at multiple levels. Transcriptomics and proteomics have revealed gene expression patterns associated with biofilm development, such as the upregulation of genes involved in surface attachment, matrix production, stress response, and quorum sensing [18,19,93], while metabolomics identifies key metabolic pathways that sustain biofilm communities [18,93]. CRISPR-based functional genomic approaches allow targeted studies of biofilm-related genes, providing mechanistic insights into their roles in host specificity and host switching [94,95]. While *V. fischeri* uses biofilms for mutualistic benefits, *V. vulnificus* biofilms enable the bacterium to evade immune responses. The different functions of biofilms highlight how bacteria can utilize and optimize biofilm phenotypes for survival, whether through cooperation with the host or to resist host defense mechanisms during pathogenesis.

## 6. Advances in Understanding Marine Biofilms

Recent research has demonstrated the adaptability of marine biofilms, highlighting microbial communities’ ability to thrive under fluctuating environmental conditions. Biofilm structure and composition exhibit dynamic alterations based on environmental factors such as temperature, salinity, and nutrient availability [8,30,69,96,97]. Environmental shifts such as climate change and human-induced habitat alterations drive biofilm evolution. These changes highlight the plasticity and ability of biofilm-forming bacteria to adapt quickly, offering us insights into microbial evolution in real time.

In marine symbiotic systems, novel regulators of biofilms continue to be identified, such as hybrid two-component systems that enable bacteria to sense and respond to host-secreted metabolites [42,50,98]. Additionally, transcriptomic analyses have uncovered species-specific regulatory networks that differentiate commensal from pathogenic biofilms, shedding light on the fine-scale genetic adaptations that dictate host interactions [19,99,100]. Mechanisms of biofilm evolution serve as incubators for microbial diversification within the biofilm community as well as the planktonic bacteria that are released from these structures. The protective and close-knit environment of biofilms enhances horizontal gene transfer, facilitating the spread of adaptive traits such as antibiotic resistance, metabolic versatility, and host colonization factors [101,102]. Studies have demonstrated that biofilm-mediated selection pressures drive the emergence of phenotypic variants with enhanced fitness, contributing to microbial evolution in both natural and host-associated settings [5,103,104]. *Vibrio* biofilms promote phase variation and genetic heterogeneity, enabling populations to withstand environmental fluctuations and host immune challenges [7]. Similar adaptive strategies are observed in other marine bacteria such as *Pseudoalteromonas* and *Shewanella*, which also form robust biofilms and exhibit regulatory plasticity in response to environmental stress. Members of the *Roseobacter* clade, abundant in marine environments, form biofilms that play essential roles in sulfur and carbon cycling and are highly responsive to changes in nutrient conditions and host-derived signals. These examples, alongside *V. fischeri*, highlight how diverse marine bacteria leverage biofilm formation as a strategy for persistence and interaction within their respective ecological niches. Understanding these evolutionary mechanisms offers insights into the persistence and resilience of *V. fischeri* within its mutualistic relationship with the squid host, revealing fundamental processes that govern microbial community dynamics and host–microbe interactions. This knowledge not only contributes to our understanding of microbial evolution and ecology at the species level but provides insight into how these systems influence broader ecosystem functions, biodiversity, and the adaptability of organisms in the face of environmental pressures.

As we continue to investigate biofilm formation, structure, and function in host-associated systems, it is imperative to include the molecular mechanisms of biofilm dispersal in mutualistic versus pathogenic *Vibrio* species, the role of interspecies interactions in biofilm architecture [5,105], and how these interactions shape stability within host-associated microbial communities. Additionally, metabolic exchange between biofilm-forming bacteria and their hosts, particularly in marine symbiotic systems, is being explored with a focus on nutrient exchange and chemical signaling [69,73]. To address these gaps, integrative approaches combining genomics, metabolomics, and live-imaging techniques are needed to better understand the dynamics of biofilm development in symbiotic relationships. The study of biofilms also has significant biotechnological implications. Engineered biofilms hold promise for applications in bioremediation, wastewater treatment, and industrial fermentation processes [106,107]. For example, disrupting quorum-sensing pathways or biofilm matrix synthesis has emerged as a promising strategy for controlling biofilm-associated pathogens [108]. By leveraging biofilm research, we can harness microbial communities for sustainable solutions while mitigating biofilm-associated challenges in both environmental and clinical settings.

## 7. Conclusions and Future Directions

The study of biofilms in *V. fischeri* and other symbiotic members of the Vibrionaceae provides critical insights into microbial ecology, host–microbe interactions, and the evolutionary dynamics of how these intricate structures evolve and persist in our environment. In marine habitats, biofilms contribute to microbial succession and the structuring of microbial communities on surfaces ranging from host tissues to substrates like sediments and ship hulls [12]. Biofilms are fundamental to the persistence of many different types of symbioses, facilitating colonization, enhancing microbial resilience, and shaping ecosystem processes [3,5,12]. Their formation enhances bacterial stability and host colonization, as observed in the structured biofilms of *V. fischeri* that mediate light organ colonization in sepiolid squid species [3,109]. Similarly, biofilms in pathogenic marine *Vibrio* species play a role in disease progression, demonstrating the dual nature of biofilm-mediated persistence [71].

Comparative analyses of biofilm strategies across *Vibrio* species reveal conserved regulatory mechanisms while highlighting species-specific adaptations. The quorum-sensing and c-di-GMP signaling pathways that regulate biofilms are shared among multiple *Vibrio* species, yet their functional outputs vary depending on the ecological niche and host interactions [110]. For example, while *V. cholerae* biofilms enhance environmental survival and transmission via waterborne routes, *V. fischeri* biofilms are tailored for long-term mutualistic colonization within squid and fish light organs [3,111]. These findings emphasize the ecological plasticity of biofilm regulation and the evolutionary pressures that shape biofilm-associated lifestyles in marine bacteria.

Despite these advancements, several key questions remain unanswered. How do biofilm-associated genetic networks evolve in response to environmental pressures? What are the long-term consequences of biofilms on host physiology and microbial community dynamics? Recent studies suggest that biofilms may drive microbial diversification through horizontal gene transfer and phase variation, yet the extent to which these processes contribute to long-term microbial adaptation in *V. fischeri* remains unclear [6,95,112]. Additionally, the interplay between host immune responses and biofilm formation requires further investigation, particularly in symbiotic systems where biofilms may actively modulate host signaling to maintain stable interactions [3,6,32]. Addressing these questions will require interdisciplinary approaches that integrate molecular biology, ecology, and computational modeling to develop a holistic understanding of biofilm dynamics.

Future research on *Vibrio* biofilms holds significant promise for applied marine microbiology. In aquaculture, promoting beneficial *Vibrio* biofilms may enhance probiotic effectiveness and contribute to pathogen resistance in farmed marine species [81]. Studies of *Vibrio* biofilms also offer insights into biofilm regulation and resilience in fluctuating marine environments, which can improve strategies for managing microbial communities under changing ocean conditions, including acidification and pollution [109,110]. These findings reflect broader themes in biofilm research, where understanding regulatory mechanisms and community dynamics can improve applications across biotechnology and environmental management [23,108].

Moreover, as tools such as single-cell imaging, real-time metabolomics, and CRISPR-based functional genomics advance, they will enable deeper explorations of biofilm heterogeneity and its implications for microbial adaptation and survival [95,113,114,115,116]. Applying these technologies in marine *Vibrio* systems will deepen our understanding of biofilm-mediated symbioses such as those between *Vibrio fischeri* and squids, supporting strategies for harnessing or controlling biofilms in diverse marine contexts. Ultimately, integrating species-specific insights from *Vibrio* with general principles of biofilm biology will strengthen both basic microbial ecology and applied environmental biotechnology.

## Figures and Tables

**Table 1:** Key host cues and bacterial genes regulating biofilm formation during *Vibrio fischeri*–*Euprymna scolopes* symbiosis.

Category	Molecule/Gene	Type/Role	Function in Biofilm or Symbiosis	Reference
Host Cue	Chitin/chitobiose	Carbohydrate	Induces chemotaxis and activates *syp* genes to initiate biofilm formation.	[46]
Host Cue	Nitric oxide (NO)	Reactive species	Temporally inhibits biofilm formation; contributes to daily symbiont expulsion.	[47]
Host Cue	Mucus (mucins)	Glycoprotein	Provides scaffold for aggregation; supports early colonization.	[48]
Host Cue	Temperature/pH shift	Environmental	Signals transition from seawater to crypts; modulates *V. fischeri* gene expression.	[49]
Regulator	*rscS*	Sensor kinase	Master regulator that initiates *syp* biofilm gene expression.	[40,50]
Regulator	*sypG*	Response regulator	Activates transcription of *syp* locus for polysaccharide production.	[51]
Regulator	*sypE*	Dual-function regulator	Modulates *sypA* activity, balancing biofilm formation and dispersal.	[51]
Matrix Genes	*sypA*–*sypR*	Structural operon	Encodes exopolysaccharide synthesis and transport for biofilm matrix.	[52]
Negative Regulator	*binK*	Sensor kinase	Inhibits *syp* transcription and biofilm formation; responds to environmental cues.	[53]
Surface Interaction	*pilA*, *pilT*	Type IV pili	Essential for initial attachment to host mucus.	[54]
Motility Gene	*flrA*, *motB*	Flagellar regulators	Loss reduces motility and promotes biofilm formation.	[55]
Second Messenger	*mifB*	c-di-GMP synthase	Modulates biofilm via intracellular signaling pathways.	[56]
Quorum Sensing	*luxR*/*luxO*	Transcriptional regulators	Coordinates biofilm gene expression and light production in response to population density.	[57]

## Data Availability

No new data were created or analyzed in this study.
